# A Promising Strain for Wheat Growth Promotion and Antifungal Activity Against Fungal Phytopathogens: *Bacillus velezensis* TRQ67

**DOI:** 10.3390/microorganisms14081825

**Published:** 2026-08-18

**Authors:** Kevin Montañez-Acosta, Amelia C. Montoya-Martínez, Ixchel Campos-Avelar, Pamela H. Morales-Sandoval, Fannie I. Parra-Cota, Lily X. Zelaya-Molina, Debasis Mitra, Gustavo Santoyo, Sergio de los Santos Villallobos

**Affiliations:** 1 Departamento de Ciencias Agronómicas y Veterinarias, Instituto Tecnológico de Sonora, 5 de Febrero 818 sur, Ciudad Obregón 85000, SO, Mexico; kevin.montanez219210@potros.itson.edu.mx (K.M.-A.); cristina_montoya14@hotmail.com (A.C.M.-M.); ixchel_campos_avelar@hotmail.com (I.C.-A.); pamesandov37@gmail.com (P.H.M.-S.); 2 Campo Experimental Norman E. Borlaug, Instituto Nacional de Investigaciones Forestales, Agrícolas y Pecuarias (INIFAP), Norman E. Borlaug Km. 12, Ciudad Obregón 85000, SO, Mexico; parra.fannie@inifap.gob.mx; 3 Laboratorio de Recursos Genéticos Microbianos, Centro Nacional de recursos Genéticos—INIFAP, Boulevard de la Biodiversidad 400, Tepatitlán de Morelos 47600, JA, Mexico; lilyzelayam@yahoo.com.mx; 4 Department of Microbiology, Graphic Era (Deemed to be University), Dehradun 248002, UT, India; debasismitra3@gmail.com; 5 Instituto de Investigaciones Químico Biológicas, Universidad Michoacana de San Nicolás de Hidalgo, Ciudad Universitaria, Edificio A1’, Morelia 58030, MC, Mexico; gustavo.santoyo@umich.mx

**Keywords:** plant growth-promotion, genomics, *Bacillus*, biological control, taxonomic affiliation

## Abstract

The rising global food demand requires boosting agricultural productivity without compromising environmental sustainability, especially in the face of intensive agrochemical use and soil degradation. Based on this, strain TRQ67 was isolated from wheat rhizosphere soil in the Yaqui Valley, Mexico, and characterized morphologically, biochemically, and genomically. Strain TRQ67 possesses a genome of 4.04 Mbp across 37 contigs with a G + C content of 46.3%, comprising 4127 coding DNA sequences (CDSs), and was identified as *Bacillus velezensis* through Overall Genome Relatedness Indices (OGRIs), including Average Nucleotide Identity (OrthoANI = 99.12%) and Genome-to-Genome Distance Calculator (Formula 2: 92.6%). The genome revealed key functional genes associated with auxin biosynthesis (*trpABCDEF* and *yhcX*), iron acquisition (*dhbABF*), nutrient solubilization (*gabD*, *acnAB* and *pyc*), stress response (*clpCEPX* and *pspA*), antifungal metabolite synthesis (*srfAABCD*, *fenABCD* and *bmyABC*), chemotaxis and motility (*cheABCD*, *motAB*, *flgBCDEF*, *swrC*), bacterial fitness (*acoABR*, *acuABC* and *budABC*), exopolysaccharide production (*epsDEFHI*), sporulation (*spo0ABEF*) and bioremediation. Predicted gene functions were supported by in vitro phenotypic assays; strain TRQ67 was able to solubilize phosphate (Solubilization Index of 4.1 ± 0.46), biosynthesize siderophores (Production Index of 1.70 ± 0.16), and produce indoles (6.52 ± 0.63 µg mL^−1^). Furthermore, this strain demonstrated antagonistic activity against phytopathogenic fungi *Fusarium languescens* and *Bipolaris sorokiniana,* resulting in reductions in fungal growth area of 87.33% and 89.28%, respectively. These antagonistic effects are consistent with the presence of Biosynthetic Gene Clusters (BGCs) encoding lipopeptides (surfactin and fengycin), polyketides (difficidin, bacillaene and macrolactin H), dipeptides (bacilysin) and siderophores (bacillibactin), as identified through antiSMASH analysis. Finally, the strain significantly improved root (27.63%) and shoot (5.82%) biomass in wheat plants under controlled conditions. These results highlight *Bacillus velezensis* TRQ67 as a promising microbial inoculant with plant growth promotion capabilities and potential antifungal activity against phytopathogenic fungi, as evidenced by strong in vitro antagonistic activity, supporting its further evaluation for sustainable agricultural practices.

## 1. Introduction

Currently, the global population stands at 8 billion and is projected to reach 10 billion by 2050, which will lead to an increase in food demand ranging from 59% to 98% [1,2]. Therefore, it is essential not only to increase food availability but also to ensure it is achieved sustainably in order to prevent the degradation of ecosystems [3]. However, the use of intensive agricultural practices, high planting densities, and excessive use of agrochemicals, in addition to drought and climate change, pose significant challenges to agricultural productivity [4]. These factors also contribute to soil erosion and physicochemical degradation, loss of microbial diversity, greenhouse gas emissions, and processes such as salinization, desertification, and eutrophication of water bodies, affecting approximately 50% of arable land [5]. Therefore, addressing food security challenges requires having healthier soils, sufficient nutrient availability, and environmental conditions that support soil biological health in order to improve crop yields [6].

Wheat is one of the most important staple grains worldwide, as it serves not only as a crucial source of calories but also of plant-based protein [7]. Globally, approximately 842 million tons of wheat are produced annually, providing food for one billion people, particularly in developing countries [8,9]. The increasing international demand has driven research efforts to enhance the quality of this cereal, a trend that began with the Green Revolution in the mid-twentieth century, led by Dr. Norman Borlaug [10]. In Mexico, the primary wheat-producing region is the Yaqui Valley, located in the northwest of the state of Sonora, where annual production reaches approximately 1.58 × 10^6^ tons, accounting for 59% of the national output [11]. However, the intensification of wheat cultivation in this region has raised concerns regarding the excessive use of agrochemicals and inefficient irrigation practices, contributing to environmental problems and the degradation of the local agroecosystem [12,13].

In this context, biotechnological tools such as microbial inoculants (or bioinoculants) have gained relevance, as they have been reported to be beneficial to soil biodiversity and can contribute to reducing the use of agrochemicals by supporting more sustainable agricultural practices [14]. These inoculants may consist of bacteria used as biological control agents (BCAs) and/or as plant growth-promoting bacteria (PGPB) through direct or indirect mechanisms [15,16]. PGPB play a crucial role in the agri-food sector, as their application helps maintain soil fertility, increase nutrient bioavailability, reduce biotic and abiotic stresses in plants, suppress plant diseases and pests, and improve both crop yield and quality [17,18]. These microorganisms are commonly found in the soil surrounding the roots, within the plant’s internal tissues, and on the surface of stems and leaves, areas known respectively as the rhizosphere, endosphere, and phyllosphere [19].

In this sense, *Bacillus* is one of the most researched microorganisms for sustainable agriculture [20,21]. The genus *Bacillus* consists of Gram-positive bacteria, widely studied and used as PGPB due to their ability to form endospores, which enhances their resistance and simplifies their isolation. Several species are commonly reported, particularly *B. subtilis* and *B. cereus*, which are commonly present in soil at concentrations ranging from 10^3^ to 10^6^ CFU g^−1^ dry soil [22]. On the other hand, the decreasing cost of DNA sequencing and advances in bioinformatics have transformed microbial bioprospecting, enabling detailed characterization of the genetic and biosynthetic potential of *Bacillus* species through genome-based approaches [23], which has facilitated the identification of genes associated with direct benefits to crops [24]. These benefits include the synthesis of phytohormones (e.g., indole-3-acetic acid [IAA], gibberellins, and cytokinins), 1-aminocyclopropane-1-carboxylic acid [ACC] deaminase production, siderophore production, nitrogen fixation through nitrogenase activity, exopolysaccharide generation, and phosphate solubilization [25,26]. Furthermore, genomic mining is essential for identifying genes related to biological control, which provide indirect benefits to plants by promoting the synthesis of secondary metabolites crucial for disease suppression. Key biological control mechanisms include: (i) the secretion of antibiotics, (ii) the production of lytic enzymes, (iii) the synthesis of cyclic lipopeptides (cLPs), (iv) siderophore production, and (v) the induction of systemic resistance (ISR) [27,28].

Moreover, genomic data support the taxonomic classification of strains through Overall Genome Relatedness Indices (OGRIs), which compare the genome of interest with closely related reference taxa. Among these, Average Nucleotide Identity (ANI), Genome-to-Genome Distance Calculators (GGDCs), and phylogenomic tree analyses are widely applied for robust species-level identification, an essential component of microbial prospecting, as it ensures the selection and characterization of promising microbial candidates for agricultural bioproducts [29,30]. Thus, the integration of genomic approaches in microbial bioprospecting not only deepens our understanding of bacterial diversity and functional potential but also facilitates the selection and development of high-performance microbial inoculants to address global food security and environmental sustainability challenges [31]. In this context, the present study aims to bioprospect the bacterial strain TRQ67 for its plant-growth promotion and biological control through genomic analyses and in vitro and in vivo experiments, with a focus on developing a high-performance microbial inoculant.

## 2. Materials and Methods

### 2.1. Bacterial Isolation, Fungal Pathogens and Growth Conditions

Strain TRQ67 was isolated from the rhizosphere of a wheat commercial field (*Triticum turgidum* subsp. *durum*) in the Yaqui Valley, Mexico (Latitude: 26° 45′–27° 33′ N. Longitude: 109° 30′–110° 37′ W), under conventional agricultural practices applying synthetic fertilizers [32]. For strain TRQ67 isolation, 10 g of a composite (1 kg from 10 points collected in 1 ha) soil sample was placed in a 250 mL Erlenmeyer flask containing 90 mL of sterile distilled water (s.d.H_2_O) (121 °C and 15 psi for 15 min) and homogenized for 1 h at 150 rpm. After homogenization, serial dilutions (1:10) were prepared to a final dilution of 10^−4^, and 100 µL of each serial dilution was inoculated on Petri dishes containing Nutrient Agar (NA) with 80 ppm of terbinafine. The inoculated Petri dishes were incubated for 72 h at 30 °C. Bacterial colonies were streaked on NA Petri dishes for purification [32]. From this assay, 51 colonies were obtained and evaluated based on morphological characteristics and relative abundance. The most abundant morphology (70%, 5 × 10^6^ Colony forming units g^−1^ dry soil), which showed typical characteristics of the genus *Bacillus*, including endospore formation and Gram-positive rod-shaped cells, was selected, purified, and designated as strain TRQ67. The isolate TRQ67 was cryopreserved at −80 °C in the microbial collection *Colección de Microorganismos Edáficos y Endófitos Nativos* (COLMENA) [33,34]. For the present study, strain TRQ67 was recovered from a glycerol stock and cultured on NA Petri dishes incubated at 30 °C for 24 h. Macro- and microscopic morphological characteristics were verified.

For biocontrol assays, two fungal phytopathogen strains from Yaqui Valley, Sonora, Mexico, which belong to the COLMENA culture collection, were selected based on their emergence and agricultural relevance: *Bipolaris sorokiniana* TPQ3, the causal agent of spot blotch in wheat (ITS GenBank accession number KX137836) [35], and *Fusarium languescens* CE2, associated with *Fusarium* wilt (TEF1 GenBank accession numbers OR168964–OR168969, and RPB2 GenBank accession number OR168973) [36]. The strains were recovered from glycerol stocks, inoculated on Potato and Dextrose Agar (PDA) dishes and incubated at 30 °C for 6 days.

### 2.2. Morphological and Biochemical Characterization

Morphological and metabolic characterization of strain TRQ67 was carried out as described below. Morphological characterization was performed based on macroscopic and microscopic observations and classified according to the principles of Bergey’s Manual of Systematic Bacteriology [37]. Colony morphology was evaluated after incubation on NA dishes at 28 °C for 72 h, considering parameters such as shape, margin, elevation, texture, and pigmentation. Microscopic characteristics were examined using light microscopy after Gram staining to determine cell shape, arrangement, and Gram reaction. Endospore formation was assessed by spore staining using the Schaeffer–Fulton method [38].

Indole production assay was evaluated with a colorimetric method based on Salkowski reagent in triplicate [39]. Strain TRQ67 (1 × 10^8^ CFU mL^−1^) was inoculated in 30 mL of nutrient broth (NB) supplemented with tryptophan (100 µg mL^−1^) and incubated at 28 °C for 5 days. After that, 1 mL of the culture was taken in Eppendorf tubes and centrifuged at 8000 rpm for 10 min. Supernatant (100 µL) was mixed in a 1:2 volume ratio with Salkowski reagent (200 µL) on an ELISA plate, then incubated for 30 min in darkness at room temperature. Absorbance reading was performed at 540 nm in a spectrophotometer for quantification.

Phosphate solubilization assay was performed in triplicate by inoculating 10 µL of strain TRQ67 (1 × 10^8^ CFU mL^−1^) on Pikovskaya medium supplemented with tricalcium phosphate as an insoluble phosphorus source and incubated at 28 °C for 5 days [32]. The presence of a white/transparent halo around the bacterial colony was indicative of phosphate solubilization.

Siderophore production assay was performed in triplicate by inoculating 10 µL of strain TRQ67 (1 × 10^8^ CFU mL^−1^) into Chrome Azurol S (CAS)-Agar medium [40], then incubated at 28 °C for 5 days. The presence of an orange-yellow halo around the bacterial colony was indicative of siderophore production. Siderophore production efficiency (SPE) and phosphate solubilization efficiency (PSE) of strain TRQ67 were assessed using the Solubilization/Production Index (SI/PI), categorized into three levels: low (<1), medium (1–2), and high (>2) [41]. The index was calculated using the following formula:

SPE = *HA*/*CA*

where:

*HA* is the area of the halo including the microbial colony (cm^2^).

*CA* is the area of the microbial colony (cm^2^).

### 2.3. Genomic Analysis and Taxonomic Affiliation

For genomic DNA extraction, strain TRQ67 was cultured in NB to obtain a concentration of 1 × 10^6^ CFU mL^−1^, following the Valenzuela-Aragon et al. (2024) [42] protocol to obtain 50 µL of high-quality DNA (~150 ng µL^−1^, 260/280 ratio of ~1.9, and 260/230 ratio of 2.1). Genomic DNA was sequenced by an Illumina NovaSeq 6000 platform (Illumina, San Diego, CA, USA) using a 250 bp paired-end protocol. Libraries were prepared using the Nextera XT Library Prep Kit (Illumina, San Diego, CA, USA) according to the manufacturer’s protocol with the following modifications: the input DNA was doubled, and the PCR elongation time was extended to 45 s. DNA quantification and library preparation were performed on a Hamilton Microlab STAR automated liquid handling system (Hamilton Bonaduz AG, Bonaduz, Switzerland). The quality of the obtained reads was analyzed by FastQC version 0.11.9 [43], and Trimmomatic version 0.30 was used to remove adapter sequences and low-quality bases [44]. *De novo* assembly was performed by SPAdes version 3.14.1, selecting the “--careful” parameter for any error correction in reads [45,46]. The alignment of the assembled contigs was ordered by Mauve Contig Mover version 2.4.0 [47], using the genome of *Bacillus siamensis* KCTC 13613^T^ (GenBank accession number GCA_000262045.1) as a genome reference, due to it having the highest 16S rRNA gene similarity (100% similarity and completeness). The assembled genome’s quality and contamination were assessed using CheckM version 1.0.18 in Kbase (www.kbase.us) and QUAST version 4.4 [48,49,50].

To retrieve the 16S rRNA gene sequence, the assembled and ordered TRQ67 genome was uploaded to the ConstEst16S tool in the EzBioCloud database (www.ezbiocloud.net); the retrieved sequence was deposited in NCBI GenBank under accession number PZ770072. Subsequently, the 16S-based ID tool (www.ezbiocloud.net) was used to identify the closest genome species related to strain TRQ67 [51]. The species delimitation cutoff value used for the 16S rRNA gene identification was >98.7%, and a Neighbor-Joining phylogenetic tree based on the 16S rRNA gene sequence of these strains was constructed. The tree was inferred using MEGA version 12.1 [52], applying the Kimura 2-parameter substitution model with 1000 bootstrap replicates to assess node support. The analysis included the 14 closest *Bacillus* type strains, using *Bacillus cereus* ATCC 14579^T^ as an outgroup. Then, the whole-genome sequence of strain TRQ67 was used to affiliate at the species level with OGRIs. ANI was calculated by the OrthoANI tool through the EzBioCloud database [53]. Pair-based algorithms on BLAST (ANIb) and MUMmer (ANIm) were performed through the JSpeciesWS (https://jspecies.ribohost.com/jspeciesws/, accessed on 15 August 2026) server to determine the average percentage of orthologous gene nucleotide sequence shared by two genomes using a cut-off value ≥95–96% [53,54]. GGDC based on BLAST (version 3.0) (https://ggdc.dsmz.de/, accessed on 15 August 2026) was used to calculate the distance between the studied strains (formula no. 2) with a ≥70% cut-off value [55]. Furthermore, a whole-genome phylogenetic tree was generated with the genomic relation between strain TRQ67 and the closest *Bacillus* type strains with the Type Strain Genome Server (TYGS) tool (https://tygs.dsmz.de/, accessed on 15 August 2026) [56].

### 2.4. Genome Mining and Functional Annotation

Genome annotation of strain TRQ67 was performed using the Rapid Annotation Subsystem Technology (RAST) server version 2.0 (https://rast.nmpdr.org, accessed on 15 August 2026) by the RASTtk pipeline [57,58]. Additionally, a complementary annotation was performed using the Rapid Prokaryotic Genome Annotation (Prokka) by Proksee to obtain a circular chromosome map [59]. For the identification of Biosynthetic Gene Clusters (BGCs) related to biological control in the genome of strain TRQ67, the Antibiotics & Secondary Metabolite Analysis Shell (antiSMASH version 8.0) (https://antismash.secondarymetabolites.org, accessed on 15 August 2026) was used with the “strict” parameter for rapid identification [45,60]. Moreover, the annotated protein sequences obtained from RAST were analyzed using the PGPT-Pred tool in the PLaBAse web server to predict functional traits linked to plant growth promotion [61].

### 2.5. In Vitro Biocontrol Assay

For the biological control assay, strain TRQ67 was evaluated against the fungal phytopathogens *B. sorokiniana* TPQ3 and *F. languescens* CE2 through a dual confrontation. For this, a 5 mm diameter disk containing fungal mycelia was placed in the center of a Petri dish with PDA, followed by incubation at 30 °C for 6 days. Afterwards, conidia were collected by adding 5 mL of s.d_._H_2_O with 0.1% Tween 80 and scraping the surface, then quantified in a hemocytometer and adjusted to 1 × 10^5^ conidia mL^−1^. In parallel, strain TRQ67 was grown in NB for 24 h at 30 °C and 120 rpm in an orbital shaker. The cell suspension was centrifuged at 4000 rpm for 10 min at 4 °C; the formed pellet was washed twice with s.d.H_2_O and adjusted to an OD of 0.5 [58]. In vitro dual assays were performed as described by Villa-Rodríguez et al. (2019) [13]. For this, 10 µL of the conidial suspension was placed at the center of each PDA dish (9 cm in diameter), and 10 µL of strain TRQ67 bacterial suspension was applied at two equidistant points from the fungal inoculum, 3 cm apart. Each treatment was performed in triplicate for each fungal phytopathogen, with its respective control treatment containing only the conidial suspension. The inoculated PDA dishes were incubated for 10 days at 25 °C. The fungal growth area was measured at day 10 through photographs of each plate using ImageJ software version 1.54p [62], and fungal inhibition was calculated according to the following equation:

Fungal Inhibition (%) = [(*FGC* − *FGB*)/*FGC*] × 100

where:

*FGC* is the fungal growth area of the control treatment (cm^2^). 

*FGB* is the fungal growth area of the bacterial strain TRQ67 treatment (cm^2^).

### 2.6. In Vivo Plant Growth Promotion Assay

To assess the plant growth promotion potential of strain TRQ67, an in vivo plant–bacteria interaction assay was conducted using the wheat (*Triticum turgidum* subsp. *durum*) CIRNO C2008 variety. For strain TRQ67 treatment, the inoculum was prepared according to the conditions previously described (see Section 2.5), with the final cell concentration adjusted to 1 × 10^8^ CFU mL^−1^ in s.d.H_2_O. For this assay, a 128-well germination tray was used. A single seed was sown per well containing 40 g of peat moss as substrate (Tierra PRO-MIX-FLEX, ICAPSA). Treatments (n = 100) were individually inoculated with 1 mL of cell suspension per well during sowing, and s.d.H2O was applied to the uninoculated treatment. Plants were irrigated every other day with 2 mL of s.d.H_2_O and maintained at 24 °C. After 20 days, the seedlings were harvested to measure shoot and root length (cm) as well as shoot and root dry weight (mg) [40].

### 2.7. Statistical Analysis

All statistical analyses were performed in STATGRAPHICS Plus Version 5.1. Differences between the control and strain TRQ67 treatment were evaluated using a two-sample *t*-test (*p* ≤ 0.05) for both growth promotion and biological control assays. Reported values correspond to the mean of replicates from independent experiments, with ± values representing standard deviation (SD).

## 3. Results

### 3.1. Morphological and Biochemical Traits of Strain TRQ67

**Figure 1:**
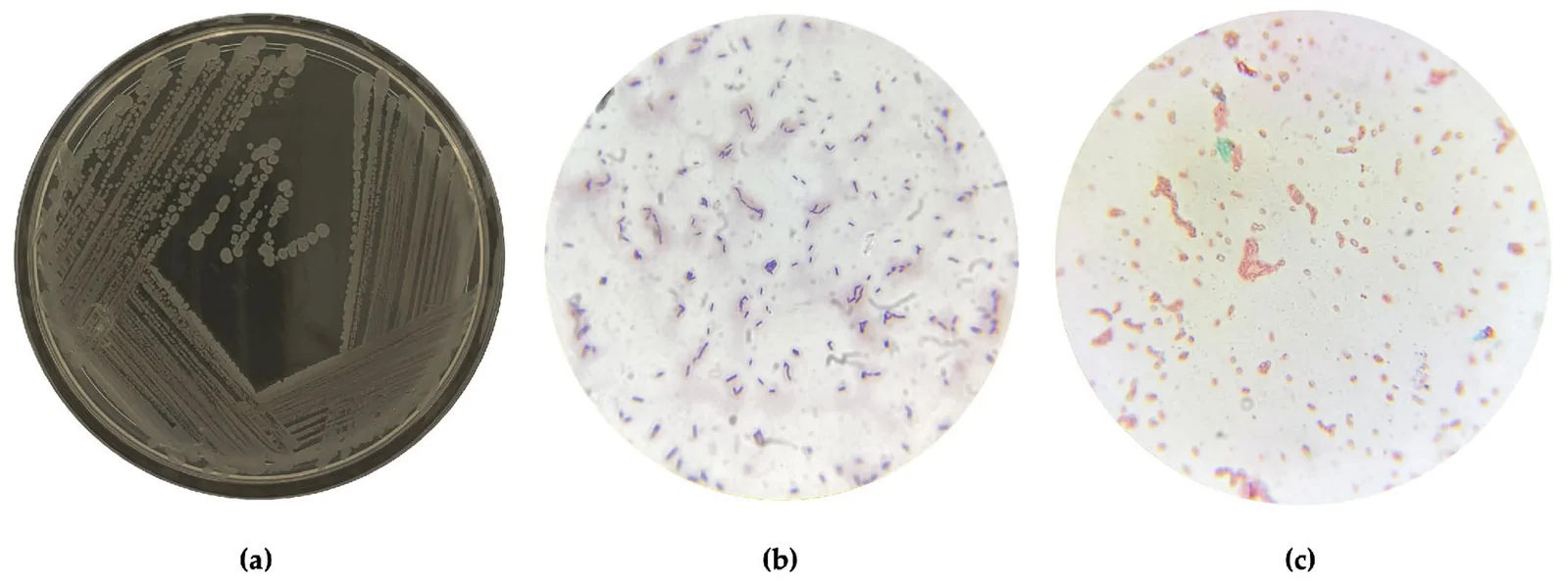
Morphological traits of strain TRQ67. (**a**) Colony morphology observed on an NA Petri dish. (**b**) Microscopic visualization of Gram-positive bacilli (100×). (**c**) Microscopic visualization of endospores through Schaeffer–Fulton staining (100×).

When grown on NA dishes, strain TRQ67 produced spreading, discrete colonies with a slimy texture, whitish pigmentation, irregular margins, and crateriform elevation (Figure 1a). Microscopic examination indicated Gram-positive rod-shaped cells, organized as single cells or in short chains (Figure 1b). Additionally, strain TRQ67 demonstrated the ability to produce endospores. Schaeffer–Fulton method revealed ellipsoidal endospores located centrally within the sporangium with slight swelling of the mother cell (Figure 1c).

In vitro metabolic assays revealed that strain TRQ67 synthesizes indole compounds at a concentration of 6.52 ± 0.63 µg mL^−1^. Also, this strain demonstrated a high phosphate solubilization efficiency of 4.1 ± 0.46 (SI). Alongside these traits, the strain exhibited siderophore production, evidenced by a yellow halo surrounding the bacterial colony with an intermediate iron-chelating activity of 1.7 ± 0.16 (PI).

### 3.2. Taxonomic Affiliation of Isolated Strain TRQ67

Based on the 16S rRNA gene sequence of strain TRQ67 and comparative analysis using the EzBioCloud database 16S-Based ID, a high similarity was revealed with 14 *Bacillus* species exceeding the cutoff value (>98.7%) for species identification. The highest identity values (100%) were observed with *Bacillus siamensis* KCTC 13613^T^ and *Bacillus velezensis* CR-502^T^, with a completeness of 100% and 95.4%, respectively (Table 1).

**Figure 2:**
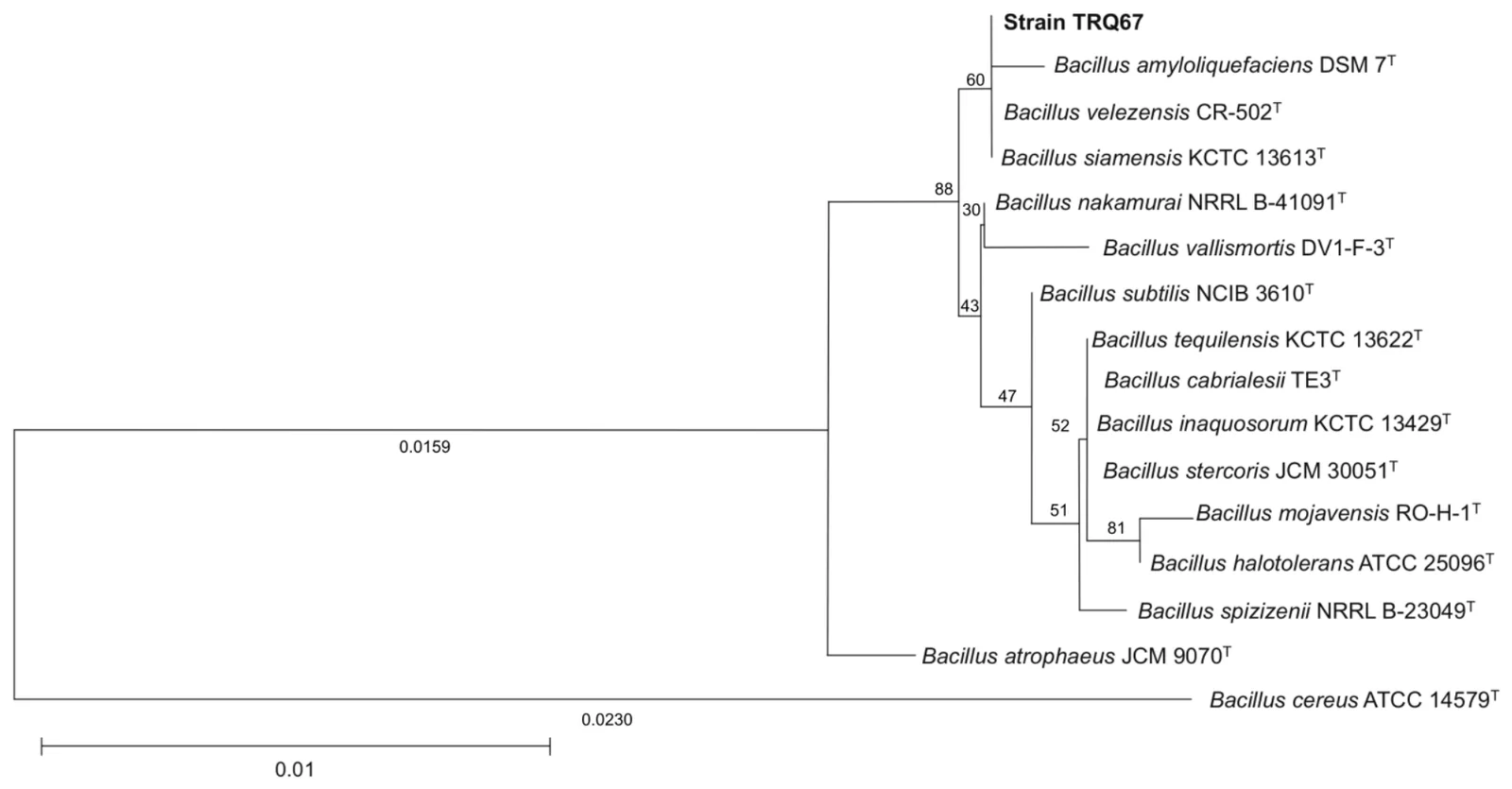
Neighbor-Joining phylogenetic tree of strain TRQ67 based on the 16S rRNA gene sequences, constructed using MEGA version 12.1 with the Kimura 2-parameter substitution model and 1000 bootstrap replicates. Bootstrap support values above 50% are shown at each node. Strain TRQ67 (GenBank accession: PZ770072) is shown in bold. *Bacillus cereus* ATCC 14579T was used as an outgroup.

The Neighbor-Joining phylogenetic tree based on the 16S rRNA gene sequences placed strain TRQ67 within the *Bacillus subtilis* species complex (Figure 2), grouping it in the same clade as *Bacillus amyloliquefaciens* DSM 7^T^, *Bacillus velezensis* CR-502^T^, and *Bacillus siamensis* KCTC 13613^T^, with a bootstrap support value of 60%. *Bacillus cereus* ATCC 14579^T^ formed a separate outgroup branch.

Species-level affiliation performed by OGRIs (Table 2), with ANI and GGDC delimited by cutoff values of ≥95–96% and ≥70%, respectively, indicated the highest genome relatedness with *Bacillus velezensis* CR-502, showing ANIb = 98.9%, ANIm = 99.2%, OrthoANI = 99.12%, and GGDC = 92.6%. These values were superior to those of the other 13 *Bacillus* species found in the EzBioCloud database, such as *B. siamensis* KCTC13613 (OrthoANI = 94.22% and GGDC = 56.3%) and *B. amyloliquefaciens* DSM 7 (OrthoANI = 94.12% and GGDC = 55.5%), which are below the established threshold.

**Figure 3:**
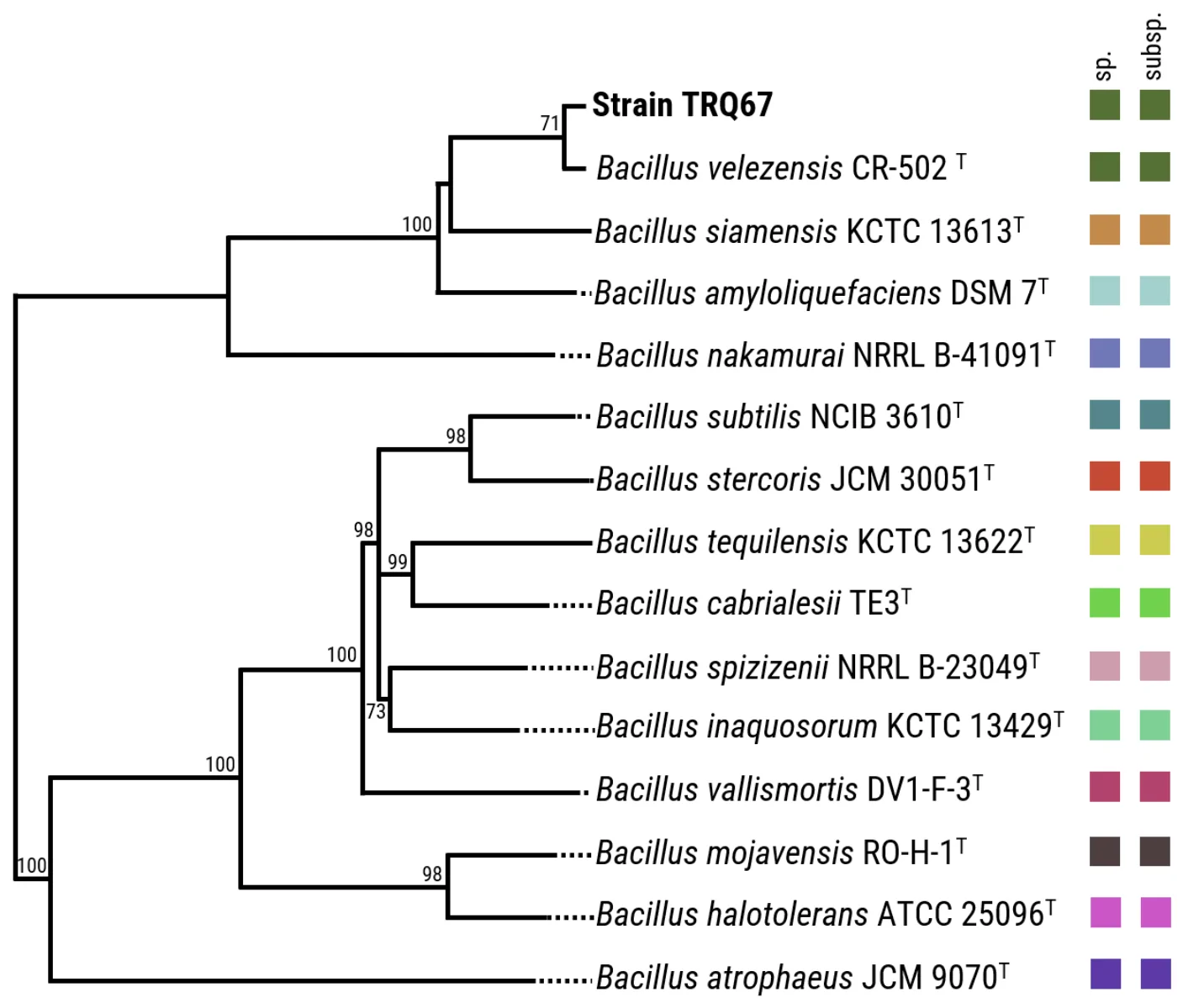
Phylogenomic relationship between strain TRQ67 and closely related species, based on genome sequences and constructed using FastMe 2.1.6.1 and Genome BLAST Distance Phylogeny (GBDP) distances (formula d5), built in TYGS. Branch lengths reflect genetic distances, and numbers at each node are pseudo-bootstrap support values (>60% from 100 replicates, average branch support of 93.7%). Square colors indicate species and subspecies groups.

In contrast to the 16S rRNA-based tree, the whole-genome-based phylogenomic tree constructed in TYGS, comparing the genome of strain TRQ67 with that of the other 14 *Bacillus* species, resolved its placement at the species level. As Figure 3 shows, strain TRQ67 was placed in the same cluster as *Bacillus velezensis* CR-502T, sharing the same clade at the species and subspecies levels, while the other closely related species were located in different clusters, in agreement with the ANI and GGDC values obtained.

### 3.3. Genome Mining and Functional Annotation of Bacillus velezensis TRQ67

**Figure 4:**
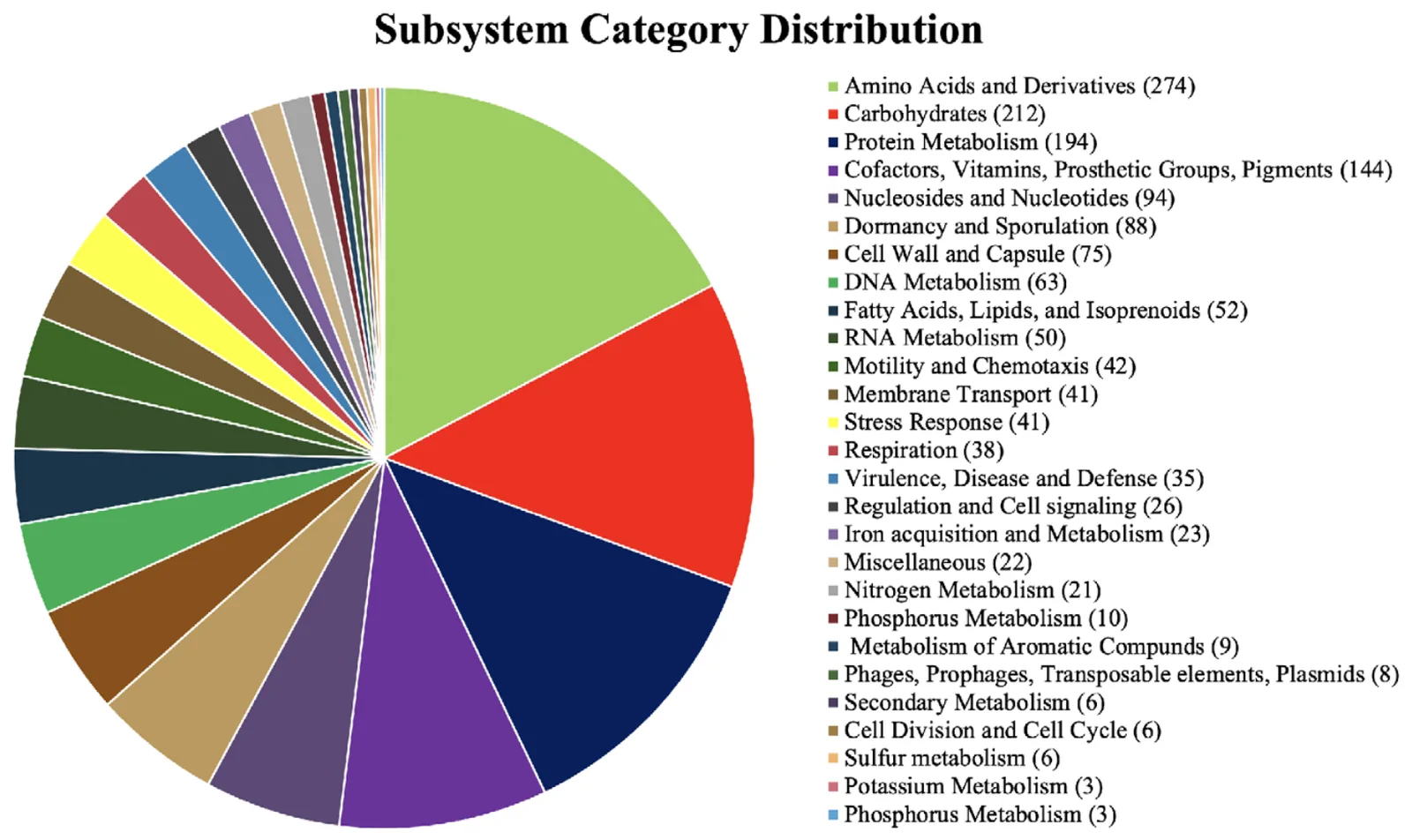
Subsystem category distribution of coding DNA sequences (CDSs) from *Bacillus velezensis* TRQ67 generated by the RASTtk pipeline (version 2.0). Numbers in parentheses indicate the number of CDSs assigned to each subsystem category. CDSs: 4127; CDSs in Subsystems: 1111; Subsystems: 321.

The draft genome assembly of strain TRQ67 consisted of 4,047,641 bp across 37 contigs with an N50 value of 612,824 bp and an L50 value of 3. Quality assessment confirmed the integrity of the assembly (99.59% completeness), and no contamination was detected. The genome possesses a G + C content of 46.3% and 4127 coding DNA sequences (CDSs) distributed in 321 subsystems according to RAST functional annotation (Figure 4). The most abundant subsystems included those related to (i) amino acids and derivatives (274 CDSs), (ii) carbohydrates (212 CDSs), (iii) protein metabolism (194 CDSs), (iv) cofactors, vitamins, prosthetic groups, and pigments (144 CDSs), and (v) dormancy and sporulation (88 CDSs). Moreover, strain TRQ67 exhibited genes associated with plant growth promotion, such as (i) iron acquisition and metabolism (23 CDSs); (ii) stress response (41 CDSs), particularly oxidative stress (12 CDSs) and osmotic stress (15 CDSs); and (iii) plant hormone production, such as auxin biosynthesis (4 CDSs). Strain TRQ67 genome also contains biological control genes related to (iv) virulence, disease, and defense (35 CDSs), including invasion and intracellular resistance (12 CDSs), bacteriocins, ribosomally synthesized antibacterial peptides (6 CDSs), and resistance to antibiotics and toxic compounds (17 CDSs).

Genome mining of *B. velezensis* TRQ67 using the antiSMASH 8.0 web server revealed the presence of seven BGCs associated with the production of secondary metabolites involved in antibacterial and antifungal traits (Table 3). These include nonribosomal peptide synthetase (NRPS) clusters responsible for the synthesis of surfactin, fengycin, and bacillibactin. Additionally, trans-AT PKS clusters involved in the biosynthesis of difficidin, bacillaene, and macrolactin H were identified. A biosynthetic gene cluster encoding the dipeptide antibiotic bacilysin was also detected and classified by antiSMASH as an “other” BGC.

**Figure 5:**
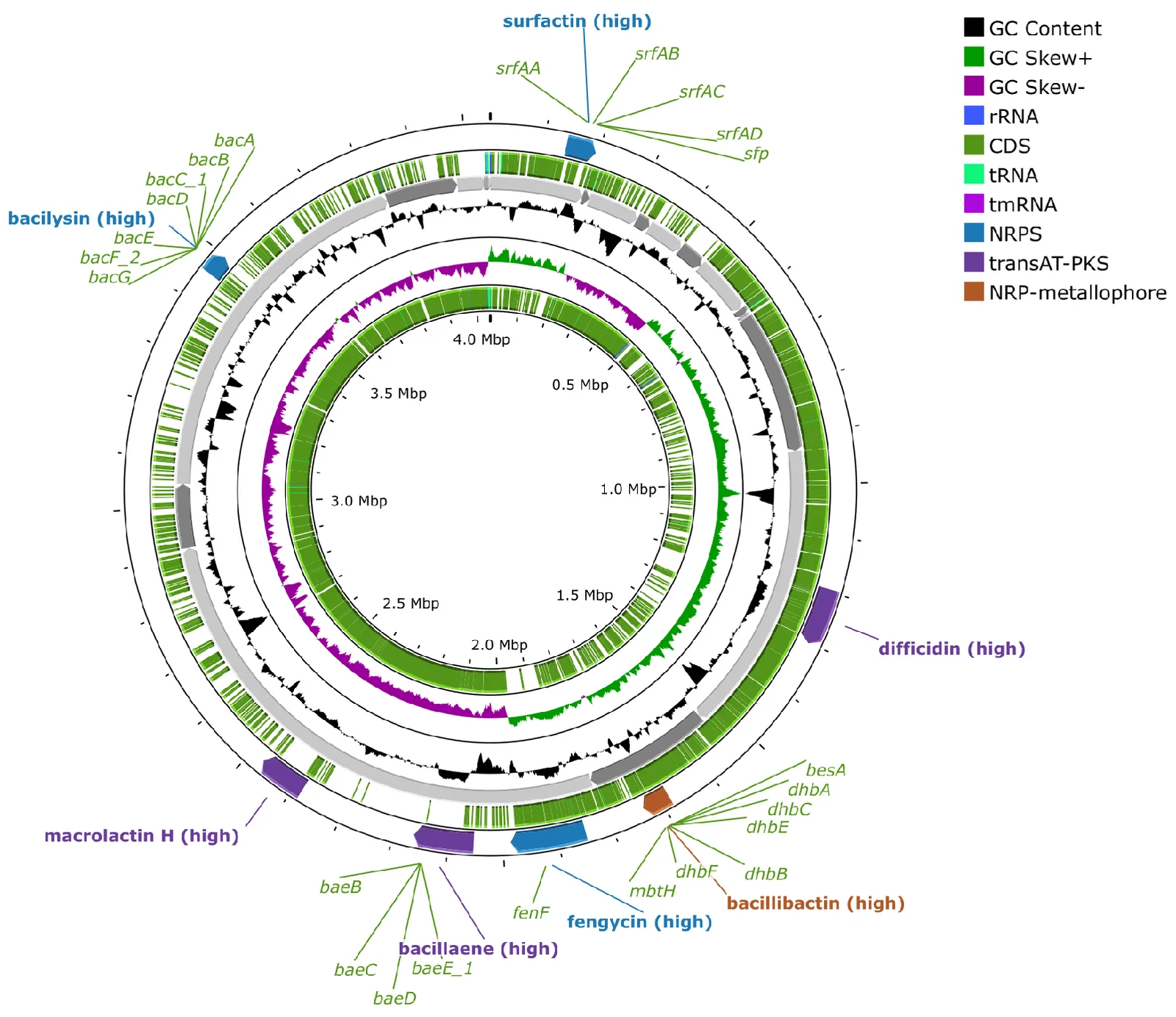
Genomic architecture and biosynthetic potential of *Bacillus velezensis* TRQ67. Circular representation of the annotated chromosome generated with Proksee, based on PROKKA annotation and BGC prediction using antiSMASH.

Complementing the genomic characterization, the circular genome map of *Bacillus velezensis* TRQ67 generated with Proksee was annotated with 4009 coding DNA sequences (CDSs), 86 tRNAs, 14 rRNAs, and 1 tmRNA, based on PROKKA annotation (Figure 5). The inner rings illustrate the GC content and GC skew, while the outer rings show the distribution of CDSs, RNA elements, and predicted BGCs identified by antiSMASH. These include NRPS and polyketide synthase (transAT-PKS) clusters, as well as NRP-metallophore types.

**Figure 6:**
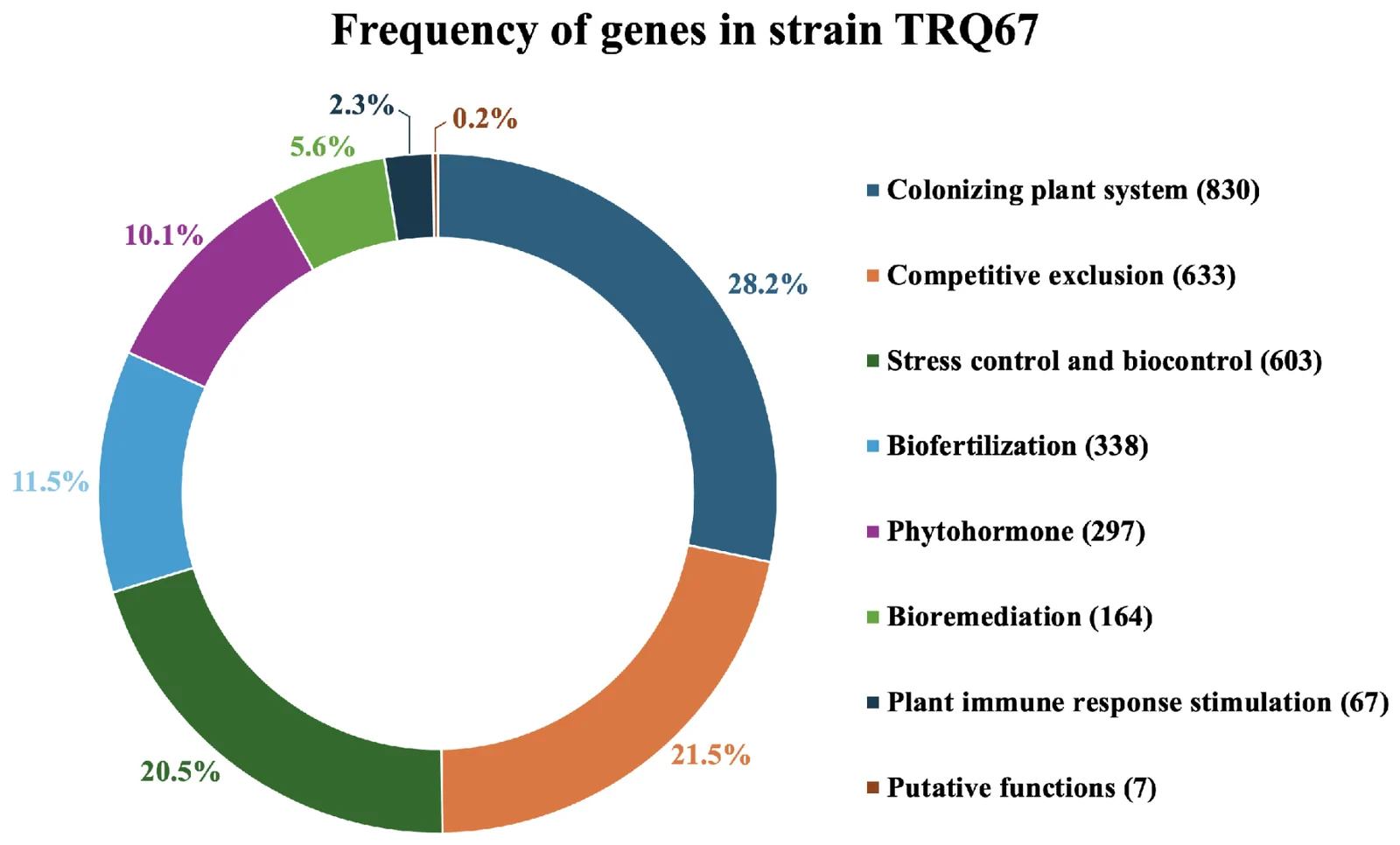
Frequency of genes involved in plant growth-promoting traits identified in the *B. velezensis* TRQ67 genome, annotated using PGPT-Pred analysis in PLaBAse. Numbers in parentheses indicate the number of genes assigned to each functional category.

Genome analysis of *B. velezensis* TRQ67 using PGPT-Pred through the PLaBAse platform identified 2939 genes associated with plant growth-promoting traits (Figure 6 and File S1), distributed across eight functional categories. The largest proportion corresponded to plant system colonization, encompassing chemotaxis genes (*cheABCD*) and flagellar motility genes (*motAB*, *flgBCDEF,* and *swrC*). The second most represented category was competitive exclusion, which included genes for compound biosynthesis (*acoABR*, *acuABC,* and *budABC*) for enhanced rhizosphere fitness, exopolysaccharide production (*epsDEFHI*), and sporulation (*spo0ABEF*). Stress control and biocontrol genes were found, including abiotic stress response genes (*clpCEPX* and *pspA*) and biosynthetic genes for antifungal lipopeptide production (*srfAABCD, fenABCD,* and *bmyABC*). The biofertilization category presented gene functions associated with iron acquisition (*dhbABF*), nutrient solubilization mediated by organic acid production (*gabD*, *acnAB,* and *pyc*), and biological nitrogen fixation (*nifMSU*). Phytohormone production genes included tryptophan biosynthesis (*trpABCDEF)* and a nitrilase involved in the IAA pathway (*yhcX*). The bioremediation category comprised genes associated with heavy metal alleviation (*zur* and *arsB*) and detoxification of organophosphate compounds (*opaA*). A smaller proportion of genes were linked to stimulation of the plant immune response and putative uncharacterized functions.

### 3.4. In Vitro Assay for Biological Control of B. velezensis TRQ67

**Figure 7:**
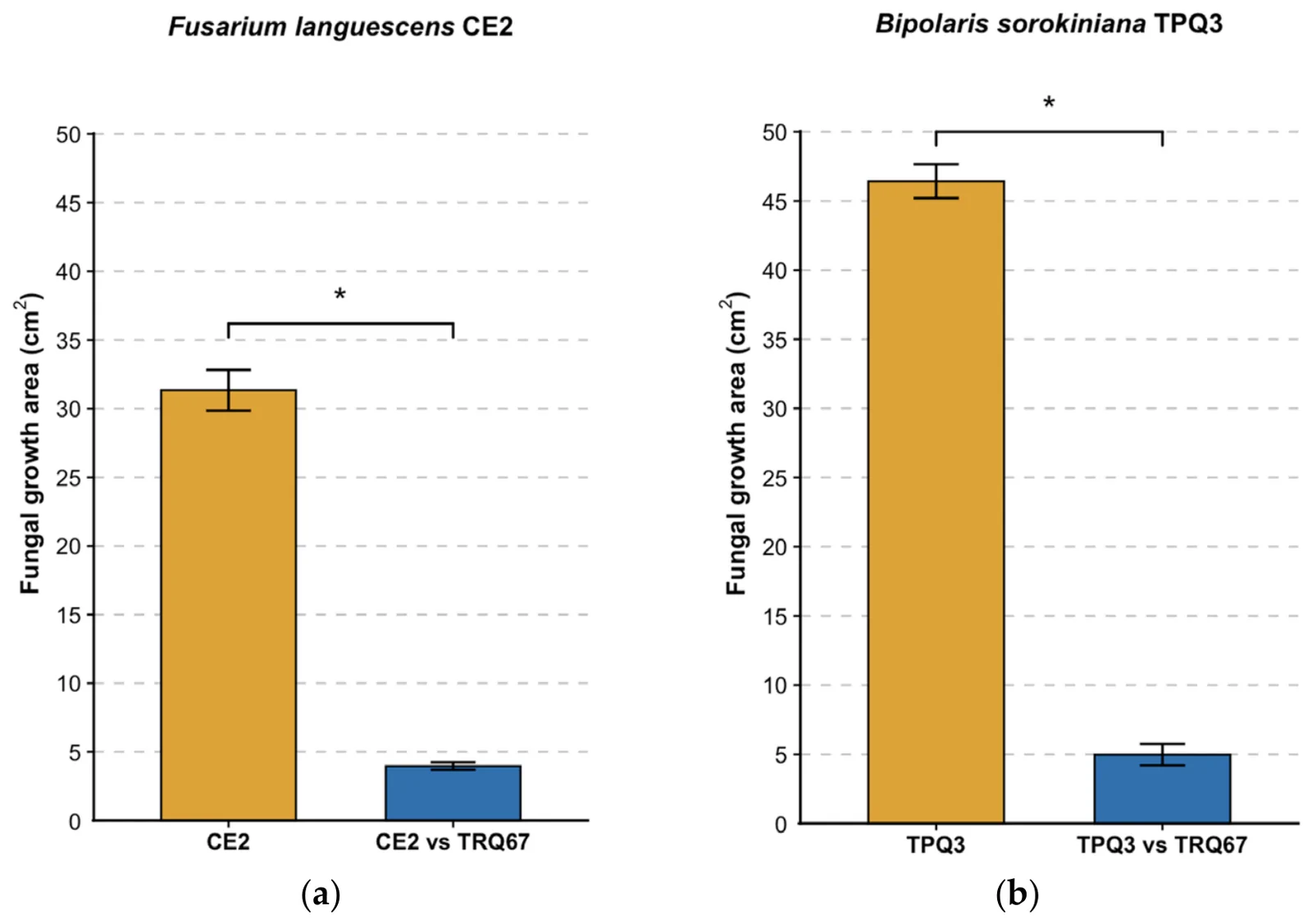
In vitro antagonistic activity of *B. velezensis* TRQ67 against phytopathogenic fungi. (**a**) *F. languescens* CE2 and (**b**) *B. sorokiniana* TPQ3 mean fungal growth area (cm^2^) ± SD under control conditions and co-cultivation with strain TRQ67 in dual confrontation assays. * indicates statistically significant differences between treatments determined by a two-sample *t*-test (*p* ≤ 0.05).

The in vitro dual confrontation assay revealed that *B. velezensis* TRQ67 possesses strong antagonistic activity against both phytopathogens evaluated. At day 10 of the confrontation assay, strain TRQ67 inhibited the growth of *F. languescens* CE2 and *B. sorokiniana* TPQ3 by 87.33% and 89.28%, respectively (Figure 7).

**Figure 8:**
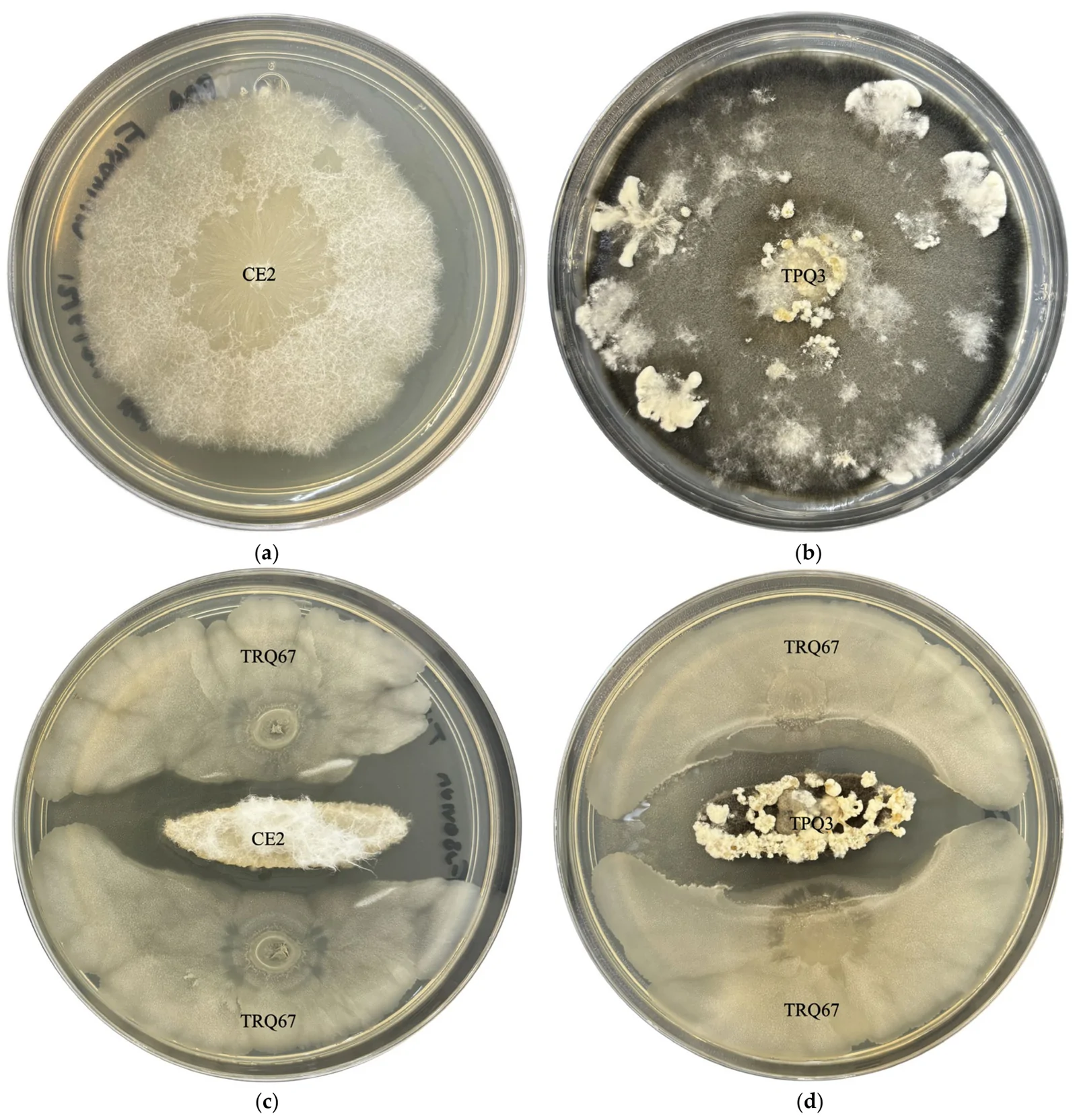
Dual confrontation assay illustrating the antagonistic interaction between *Bacillus velezensis* TRQ67 and phytopathogenic fungi, showing strong inhibition, altered fungal growth, visible inhibition zones, and bacterial swarming expansion. (**a**) *F. languescens* CE2 control. (**b**) *B. sorokiniana* TPQ3 control. (**c**) TRQ67 vs. *F. languescens* CE2. (**d**) TRQ67 vs. *B. sorokiniana* TPQ3.

The antagonistic effect of strain TRQ67 was evident upon observation of the dual confrontation assay. In the absence of TRQ67 (Figure 8a,b), both fungi exhibited unrestricted radial growth, forming dense and well-developed mycelial masses. In contrast, when co-cultivated with strain TRQ67 (Figure 8c,d), fungal development was significantly suppressed, with defined inhibition zones and morphological alterations at the contact interface. Moreover, the swarming behavior of strain TRQ67 colonies was remarkably enhanced during the antagonism assay, expanding laterally beside fungal colonies, forming large, diffused margins.

### 3.5. In Vivo Plant Growth Promotion of B. velezensis TRQ67

Plant growth promotion assay demonstrated that inoculation with *B. velezensis* TRQ67 increased plant biometric parameters with a significant difference in root length (19.08%), shoot dry weight (5.82%), and root dry weight (27.63%) compared to the non-inoculated control (Table 4), thus indicating a stimulatory effect on biomass accumulation. In contrast, shoot length showed a statistically significant decrease (3.66%) compared to the control.

## 4. Discussion

Bioprospection of PGPB through omics techniques has transformed microbial research by improving both functional and taxonomic characterization [63]. While genome mining tools facilitate the identification of BGCs and metabolic pathways relevant to agriculture [64,65], OGRIs provide accurate species-level affiliation in cases where conventional methods are insufficient [66]. In this context, whole-genome sequencing of strain TRQ67 provided detailed insights into both its genetic repertoire and taxonomic affiliation, confirming its identity and supporting its potential use in sustainable agriculture.

Strain TRQ67 exhibited morphological traits typical among soil-dwelling *Bacillus* species, which are commonly found in both natural and human-impacted soil ecosystems [67]. *Bacillus* species are also known for their adaptability to thrive in complex environments, colonize plant roots, persist under stressful conditions, and form resistant spores [2,68]. Strain TRQ67 demonstrated the ability to produce indole compounds, which are frequently associated with auxin-related activity and root system development, including enhanced root length, lateral root formation, and root hair density, thereby improving nutrient and water uptake [12,69]. Indole production is a common trait among rhizospheric bacteria, and studies suggest that there is a correlation between increased indole production by *Bacillus* species and improved stress tolerance in plants [12]. Quantification assays revealed that strain TRQ67 produces 6.52 ± 0.63 µg mL^−1^ of indole compounds, a concentration comparable to that of other rhizospheric bacterial strains from the Yaqui Valley, such as *B. cabrialesii* TE3^T^ (3.7 µg mL^−1^) and *Priestia megaterium* TRQ8 (12 µg mL^−1^), with reported values ranging from 1.5 to 48.9 µg mL^−1^ [32,40,45,58]. Indole production was evaluated in a medium supplemented with tryptophan (100 µg mL^−1^), at a concentration higher than that typically found in the rhizosphere. Therefore, the measured values reflect the biosynthetic potential of strain TRQ67 under laboratory conditions, whereas in situ production remains to be evaluated. Phosphate solubilization is another critical trait exhibited by strain TRQ67, demonstrated by a clear halo zone on Pikovskaya’s medium (SI of 4.1 ± 0.46). This value is notably higher than that reported for other *Bacillus* isolates from the Yaqui Valley, which displayed intermediate activity (SI: 1.2–1.4); furthermore, only 31% (67 of 216) of previously characterized soil strains from the region demonstrated phosphate solubilization, generally with lower efficiency than strain TRQ67 [32,70]. This process enhances the bioavailability of phosphorus, a macronutrient often present in soils in insoluble forms [71]. The solubilization mechanism is typically mediated by the secretion of organic acids and phosphatases that lower the local pH, leading to the release of phosphate from mineral complexes [72]. In addition, strain TRQ67 demonstrated a strong ability to produce siderophores, as evidenced by the formation of an orange halo on CAS medium. Siderophores are low-molecular-weight compounds (<2000 Da) that chelate ferric iron (Fe^+3^) from the environment, facilitating its uptake by bacterial cells and host plants, especially under iron-limiting conditions [73,74]. In the Yaqui Valley, *Bacillus* isolates have demonstrated varying capacities for siderophore production. For instance, Ibarra-Villarreal et al. (2023) reported Siderophore Production Index (PI) values ranging from 1.2 to 1.8 among *Bacillus* strains, positioning strain TRQ67 (PI: 1.70 ± 0.16) within this spectrum [32]. Similar trait concordance has been observed in other reported *B. velezensis* strains, such as GH1-13 (IAA production) [75], AR1 (phosphate solubilization) [76], and MBTDLP1 (siderophore production) [77]. The combined production of indole compounds, phosphate solubilization, and siderophore synthesis reflects a multifunctional strategy characteristic of effective rhizobacteria, in which hormonal modulation and improved nutrient acquisition act as complementary mechanisms that enhance nutrient availability and may explain the positive responses observed in plant assays [78].

Genetic analyses revealed that the 16S rRNA gene of strain TRQ67 showed 100% identity to reference sequences of *B. siamensis* KCTC 13613^T^ and *B. velezensis* CR-502^T^. Consistently, the Neighbor-Joining phylogenetic tree grouped strain TRQ67 with *B. velezensis* CR-502^T^, *B. siamensis* KCTC 13613^T^, and *B. amyloliquefaciens* DSM 7^T^ within the same clade, reflecting the well-documented limitation of the 16S rRNA gene to discriminate between closely related species. However, genomic analyses using OGRIs, including ANI and GGDC, clarified these taxonomic ambiguities. OGRIs strongly supported the taxonomic affiliation of strain TRQ67 as *B. velezensis*, based on the obtained OrthoANI and GGDC values of 99.12% and 92.6%, respectively. In accordance with OGRI analyses, the phylogenomic tree (via TYGS) placed the strain TRQ67 genome in the *B. velezensis* cluster.

*Bacillus velezensis* TRQ67 parameters such as draft genome size (4.04 Mbp), G + C content (46.3%) and gene number (4127 genes) are found within the typical range (3.81–4.32 Mb, 45.78% to 46.80% of G + C and 3610–4436 genes) for this species; however, it encodes more CDSs than some relatives [79], which likely reflects variations in sequencing and assembly quality and strategies [80]. RAST annotation of strain TRQ67 revealed a broad core-metabolism profile comparable to that of other *B. velezensis* strains. For instance, *B. velezensis* B26 carries major subsystems for carbohydrate metabolism (216 CDSs), stress response (43 CDSs), and virulence, disease, and defense (35 CDSs) [81]. Likewise, a pan-genomic study of over 46 global *B. velezensis* isolates reported that the most abundant CDS categories are amino acids and derivatives, transport and metabolism, carbohydrate transport and metabolism, and motility [79,82].

*Bacillus velezensis* is known for its rich arsenal of antibiotic and surfactant biosynthetic genes [83]. The antiSMASH analysis of strain TRQ67 revealed NRPS clusters for the lipopeptides (LPs) surfactin and fengycin, alongside polyketide synthase (PK) clusters for difficidin, bacillaene and macrolactin H, as well as the ribosomal peptide bacilysin and the bacillibactin siderophore cluster. LPs are amphiphilic molecules composed of a hydrophobic fatty acid linked to a hydrophilic peptide that reduce surface and interfacial tension across oil–water, air–water, and liquid–solid interfaces [84,85]. In *Bacillus,* surfactin and fengycins are among the most potent LPs, disrupting fungal membranes through pore formation and triggering cell death [86,87,88]; their production has been documented across diverse *B. velezensis* strains and conditions [89,90]. PKs, including difficidin, bacillaene, macrolactin, and the dipeptide bacilysin, represent an equally important class of antimicrobial secondary metabolites widely encoded within the *B. subtilis* group [91,92]. Additionally, bacillibactin, a catecholate siderophore, chelates Fe^3+^ under iron starvation conditions, depriving possible pathogens of this essential micronutrient and enhancing bacterial colonization [93,94]. Bacillibactin-mediated iron sequestration has been shown to contribute to biocontrol efficacy in *B. velezensis*, as strains deficient in bacillibactin biosynthesis exhibit significantly reduced antagonistic activity against phytopathogens, suggesting that this mechanism may similarly contribute to the antagonism observed against *F. languescens* CE2 and *B. sorokiniana* TPQ3 in the present study [95,96].

Functional genes of strain TRQ67, classified by PGPT-Pred, encompass eight functional categories, each supported by specific genes whose roles have been characterized in *Bacillus* and related taxa. Within the colonization of plant system category, chemotaxis-related gene (*cheABCD*) and flagellar assembly genes (*motAB*, *flgBCDEF*) support direct root colonization and surface motility, while *swrC* encodes a surfactin transporter involved in enhanced surfactin secretion to the cell surface, facilitating swarming [97,98,99].

In the competitive exclusion category, genes for acetoin-related compound biosynthesis (*acoABR*, *acuABC*, *budABC*) allow the strain to utilize alternative carbon sources and produce 2,3-butanediol, conferring enhanced rhizosphere fitness against competing microorganisms [100,101,102]. Additionally, *epsDEFHI* and *spo0ABEF* are involved in exopolysaccharide biosynthesis and master regulation of sporulation initiation, respectively, both associated with biofilm formation and long-term soil persistence [103,104,105].

Within the phytohormone production category, *trpABCDEF* and *yhcX* encode enzymes involved in IAA biosynthesis routes through the tryptophan-dependent pathway [106,107]. In the biofertilization category, *dhbABF* encodes the bacillibactin biosynthetic pathway responsible for catecholate-type siderophore assembly [69]; additionally, *gabD*, *acnAB* and *pyc* are associated with the biosynthesis of succinic, citric and malic acids, respectively [108,109,110], which are organic acids known to solubilize insoluble mineral-bound nutrients in soil through pH reduction and chelation [111]. Furthermore, *nifMSU* accessory genes associated with the nitrogen fixation machinery were identified in the genome of strain TRQ67 [112]; however, nitrogenase activity was not experimentally evaluated in the present study and should be assessed in future work to fully characterize the nitrogen fixation potential of this strain. These predicted functions are consistent with the phenotypic traits observed in vitro; however, direct gene-level validation would require targeted approaches such as gene expression analyses or mutant studies [113]. Collectively, these mechanisms likely contribute to the enhanced plant growth promotion observed in this study. Also, this distribution of categories is similar to that of other *B. velezensis* strains; for instance, *B. velezensis* BN had 26% of genes related to the colonization system, 22% to competitive exclusion, and 22% to stress control [114]. Likewise, a comparative study of tomato-associated strains found that all *Bacillus* genomes were dominated by colonization-related genes, followed by stress control and competitive exclusion traits [115].

In the stress control and biocontrol category, *clpCEPX* encodes proteases responsible for cellular protein quality control through refolding or degradation of damaged proteins under stress conditions [116,117], while *pspA* participates in cell envelope stress-sensing signal transduction [118], collectively supporting the persistence of strain TRQ67 under the arid and saline soil conditions characteristic of the Yaqui Valley [119]. Also within this category, *srfAABCD*, *fenABCD,* and *bmyABC* encode the NRPS machinery for surfactin, fengycin, and bacillomycin biosynthesis, respectively, three lipopeptide families with documented antifungal mechanisms [85]. Bacillomycin D, encoded by *bmyABC* and identified by PGPT-Pred, further broadens the antifungal repertoire of *B. velezensis* TRQ67 [83]. Furthermore, the predicted antiSMASH-identified BGCs for surfactin and fengycin may account for the strong antifungal activity observed against *F. languescens* CE2 and *B. sorokiniana* TPQ3. In dual confrontation assays, *Bacillus velezensis* TRQ67 exhibited strong antagonistic activity against two important fungal phytopathogens relevant to agriculture in the Yaqui Valley. Strain TRQ67 strongly inhibited (89%) the growth of *Bipolaris sorokiniana* TPQ3, the agent responsible for spot blotch in wheat, an emerging threat to regional wheat production [13]. This inhibition rate is comparable to that of *B. velezensis* strain BV01, with reported antagonism against *B. sorokiniana* (80%) [120]; other remarkable examples of *B. sorokiniana* inhibition were caused by strains HeN-7 (96%) [121] and NEAU-242-2 (61%) [122].

Additionally, strain TRQ67 inhibited *Fusarium languescens* CE2 by 87%, a member of the *Fusarium oxysporum* species complex (FOSC) associated with wilt in jalapeño peppers [36]. Although no prior reports link *B. velezensis* specifically to *F. languescens* antagonism, strains such as B-36 [123] and BA [36] have shown inhibition against other FOSC members of 74% and 60%, respectively, suggesting a broader antifungal potential within the species complex. However, the antagonistic activity reported here was demonstrated exclusively under in vitro conditions; therefore, *in planta* and field assays are required to confirm the antifungal potential of strain TRQ67 under plant conditions.

As mentioned previously, lipopeptides and polyketides may contribute to the observed antifungal activity through interference with cell wall integrity [124,125]. Iron sequestration through siderophore production by strain TRQ67 may enhance competitive fitness in the rhizosphere while simultaneously restricting iron availability to phytopathogens [126]. Together, these mechanisms provide a plausible genomic-based explanation for the antagonistic activity observed against both phytopathogens; however, metabolite production was not experimentally confirmed, and metabolomics analyses would be required to directly link specific compounds to the antifungal activity reported.

Moreover, strain TRQ67 exhibited highly dynamic swarming motility in confrontation assays. This swarming behavior, as a result of a coordinated multicellular movement driven by rotating flagella and the secretion of surfactin to reduce surface tension, has been closely associated with enhanced biocontrol efficacy in *Bacillus* spp. [127,128]. In strain TRQ67, this is supported by the presence of genes involved in flagellar motility and surfactin biosynthesis, which are linked to plant colonization. Importantly, this behavior appeared to be enhanced upon co-cultivation with fungal colonies, rather than during standard growth conditions, suggesting that TRQ67 may activate this mechanism as a targeted response to pathogen presence, thereby facilitating rapid surface colonization and metabolite dispersal [129,130]. Native *Bacillus* strains from the Yaqui Valley have demonstrated comparable biocontrol capacities, including antagonism against *B. sorokiniana* by *B. cabrialesii* TE5 (67%), TE3^T^ (98%), and *B. inaquosorum* TSO22, as well as against *F. languescens* CE2 by *B. cabrialesii* subsp. *tritici* TSO2^T^ (61%) [2,131].

Species of the genus *Bacillus* are well known for their ability to colonize the rhizosphere and promote plant growth through the biosynthesis of bioactive metabolites associated with mineral solubilization, metal chelation, and phytohormone production [120,132]. This is consistent with the results of strain TRQ67, which exhibited indole compound production, likely related to the enhanced root growth and biomass observed.

In plant growth promotion assays, *Bacillus velezensis* TRQ67 significantly stimulated biometric parameters, particularly those associated with root development, increasing root length (19.08%), shoot dry weight (5.82%), and root dry weight (27.63%). Although shoot length showed a statistically significant reduction of 3.66%, this was accompanied by an increase in shoot dry weight, indicating denser and stronger shoot tissue rather than impaired development [133]. This growth pattern is consistent with a dose-dependent effect of bacterial auxin production, which promotes root biomass accumulation while potentially moderating shoot elongation [134,135,136]. This growth pattern may be advantageous during early plant development, due to a compact but robust shoot with an expanded root system that improves water and nutrient acquisition capacity, enhances soil anchoring, and confers greater resistance to lodging and drought stress [137,138]. While an elevated root-to-shoot ratio is often associated with nutrient stress and reduced overall biomass [139,140], strain TRQ67 promoted simultaneous increases in root development and both root and shoot dry weights, indicating a positive effect on early plant growth [141]. Nevertheless, long-term trials evaluating later developmental stages would be needed to determine whether early growth benefits persist and translate into broader agronomic outcomes.

These results are consistent with plant growth-promoting traits reported in other *B. velezensis* strains; for instance, strain BV01 increased wheat plant height (10%) and dry weight (5%) while also enhancing leaf width and shoot thickness in peppers (9%) [120], and strain XT1 promoted substantial increases in biometric parameters ranging from 53% to 129% across tomato, pepper, pumpkin, and cucumber [142]. From a regional perspective, native *Bacillus* strains from the Yaqui Valley, including *B. cabrialesii* TE3^T^ and consortia with *B. paralicheniformis* TRQ65 and *P. megaterium* TRQ8, have demonstrated comparable growth-promoting capabilities in wheat, with biometric improvements ranging from 25–72% [40,45,58,143].

Finally, the commercial development of *B. velezensis*-based products further supports the biotechnological relevance of this species. Notable examples include RhizoVital^®^ (*B. velezensis* FZB42, Germany), used to control soilborne diseases including *Rhizoctonia solani*; Kodiak™ (*B. velezensis* GB03, USA), registered for antagonistic activity against *Fusarium oxysporum* and *R. solani*; Botrybel (Spain), commercialized for control of *Botrytis cinerea*; and Fungifree AB™ (*B. velezensis* 83, Mexico), marketed as a foliar biofungicide against *B. cinerea* [82,144,145,146], reinforcing the broad biotechnological potential of the species and supporting the promise of *B. velezensis* TRQ67 as a high-performance bioinoculant.

## 5. Conclusions

The comprehensive evaluation of strain TRQ67 through in vitro and in vivo assays, supported by genomic analysis and taxonomic confirmation, demonstrated its plant growth-promoting ability and antifungal potential against phytopathogenic fungi. Whole-genome sequencing and OGRI analyses confirmed its identity as *Bacillus velezensis*, while gene mining revealed a diverse set of plant-beneficial functions, including stress response mechanisms and biosynthetic gene clusters for antifungal metabolites. In vitro, TRQ67 exhibited key plant growth-promoting traits, including phosphate solubilization, siderophore production, and indole compound synthesis, along with strong antagonistic activity against *Fusarium languescens* and *Bipolaris sorokiniana*. Plant growth-promotion trials confirmed its positive effects on wheat development, showing significant improvements in root and shoot biomass.

These results confirm the dual functionality of strain TRQ67 and support its potential as a high-performance microbial inoculant for sustainable agricultural systems. Future work should focus on evaluating its effectiveness under field conditions, improving its formulation for long-term stability, and analyzing its ecological interactions in soil to strengthen its application in real agricultural practices.

## Figures and Tables

**Table 1:** 16S rRNA gene similarity percent (>98.7%) obtained from the EzBioCloud database.

Taxon Name	Type Strain	16S rRNA Accession	Similarity (%)	Completeness (%)
*Bacillus siamensis*	KCTC 13613^T^	AJVF01000043	100	100
*Bacillus velezensis*	CR-502^T^	AY603658	100	95.4
*Bacillus amyloliquefaciens*	DSM 7^T^	FN597644	99.9	100
*Bacillus nakamurai*	NRRL B-41091^T^	LSAZ01000028	99.9	100
*Bacillus subtilis*	NCIB 3610^T^	ABQL01000001	99.79	100
*Bacillus vallismortis*	DV1-F-3^T^	JH600273	99.69	100
*Bacillus tequilensis*	KCTC 13622^T^	AYTO01000043	99.69	100
*Bacillus cabrialesii*	TE3^T^	MK462260	99.69	100
*Bacillus inaquosorum*	KCTC 13429^T^	AMXN01000021	99.69	100
*Bacillus stercoris*	JCM 30051^T^	MN536904	99.69	100
*Bacillus atrophaeus*	JCM 9070^T^	AB021181	99.59	100
*Bacillus halotolerans*	ATCC 25096^T^	LPVF01000003	99.59	100
*Bacillus spizizenii*	NRRL B-23049^T^	CP002905	99.59	100
*Bacillus mojavensis*	RO-H-1^T^	JH600280	99.48	100

**Table 2:** OGRI-based taxonomic affiliation of strain TRQ67, determined by ANI and GGDC values.

Taxon Name	Type Strain	GenBank Accession	ANIb (%)	ANIm (%)	OrthoANI	GGDC (Formula 2)
*Bacillus velezensis*	CR-502^T^	GCA_001461825.1	98.9	99.2	99.12	92.6
*Bacillus siamensis*	KCTC 13613^T^	GCA_000262045.1	93.72	94.44	94.22	56.3
*Bacillus amyloliquefaciens*	DSM 7^T^	GCA_000196735.1	93.34	94.21	94.12	55.5
*Bacillus nakamurai*	NRRL B-41091^T^	GCA_001584325.1	85.61	87.1	86.63	30.8
*Bacillus tequilensis*	KCTC 13622^T^	GCA_000507145.1	76.68	84.49	77.37	20.7
*Bacillus atrophaeus*	JCM 9070^T^	GCA_001584335.1	76.56	83.79	77.34	20.6
*Bacillus inaquosorum*	KCTC 13429^T^	GCA_003148415.1	76.54	84.38	77.49	20.9
*Bacillus mojavensis*	RO-H-1^T^	GCA_014499005.1	76.53	84.03	77.1	20.4
*Bacillus halotolerans*	ATCC 25096^T^	GCA_001637525.1	76.49	84.17	77.5	20.6
*Bacillus spizizenii*	NRRL B-23049^T^	GCA_000227465.1	76.39	84.03	77.19	20.7
*Bacillus stercoris*	JCM 30051^T^	GCA_000738015.1	73.34	84.15	77.35	20.4
*Bacillus vallismortis*	DV1-F-3^T^	GCA_004116955.1	76.3	84.03	77.13	20.4
*Bacillus cabrialesii*	TE3^T^	GCA_004124315.1	76.26	84.18	77.24	20.5
*Bacillus subtilis*	NCIB 3610^T^	GCA_000009045.1	76.17	84.25	77.11	20.6

**Table 3:** BGCs associated with biological control activity identified in the *B. velezensis* TRQ67 genome by the antiSMASH 8.0 web server.

Region	BGCs Type *	From	To	Most Similar Known Cluster	Similarity Confidence **
1.1	NRPS	140,209	195,571	surfactin	High
12.1	transAT-PKS	264,487	370,666	difficidin	High
13.1	NRP-metallophore, NRPS	140,358	192,094	bacillibactin	High
14.2	NRPS, betalactone	39,485	177,344	fengycin	High
14.3	transAT-PKS, NRPS, T3PKS	244,780	354,879	bacillaene	High
14.4	transAT-PKS	575,024	663,254	macrolactin H	High
16.2	Other	410,673	452,091	bacilysin	High

* Other BGC types refer to secondary metabolite-related proteins that do not fit into any other category of Protocluster Types from the antiSMASH 8.0 web server [60]. ** Similarity confidence “high” denotes ≥ 75% sequence identity to a characterized reference cluster.

**Table 4:** Effect of *Bacillus velezensis* TRQ67 on growth parameters of wheat (*Triticum turgidum* subsp. *durum*) in an in vivo assay.

	Shoot Length (cm)	Root Length (cm)	Shoot Dry Weight (mg)	Root Dry Weight (mg)
Control	20.95 ± 1.44	6.32 ± 1.39	20.21 ± 2.34	34.85 ± 6.18
Strain TRQ67	20.18 ± 1.89	7.52 ± 1.02	21.39 ± 2.54	44.48 ± 9.44
Percentage	−3.66%	+19.08%	+5.82%	+27.63%
*p*-value	*p* < 0.0001 *	*p* < 0.0001 *	*p* = 0.0018 *	*p* < 0.0001 *

Values represent mean ± SD of 128 plants per treatment, in triplicate. Statistical differences between treatments were determined using a two-sample *t*-test (*p* ≤ 0.05). * indicates a significant difference.

## Data Availability

The draft genome sequence has been deposited in DDBJ/ENA/GenBank under accession number JBCJFH000000000.1. The version described in this paper is the first version, under BioProject number PRJNA1080047 and BioSample number SAMN41095874. The raw data files may be consulted under the following accession number SRX24379669 at the following link: https://www.ncbi.nlm.nih.gov/sra/SRX24379669[accn], accessed on 21 June 2026.

## Supplementary Materials

The following supporting information can be downloaded at https://www.mdpi.com/article/10.3390/microorganisms14081825/s1, File S1: Complete PGPT-pred functional genome annotation of *B. velezensis* TRQ67 obtained through the PLaBAse web server.

## Abbreviations

The following abbreviations are used in this manuscript:

ACC	1-aminocyclopropane-1-carboxylic acid
ANI	Average Nucleotide Identity
ANIb	Average Nucleotide Identity based on BLAST
ANIm	Average Nucleotide Identity based on MUMmer
BCA	Biological Control Agent
BGC	Biosynthetic Gene Cluster
CAS	Chrome Azurol S
CDS	Coding DNA Sequence
CFU	Colony Forming Unit
cLP	Cyclic Lipopeptide
DNA	Deoxyribonucleic Acid
FOSC	*Fusarium oxysporum* Species Complex
GBDP	Genome BLAST Distance Phylogeny
GGDC	Genome-to-Genome Distance Calculator
IAA	Indole-3-acetic acid
ISR	Induction of Systemic Resistance
ITS1	Internal Transcribed Spacer 1
NA	Nutrient Agar
NB	Nutrient Broth
NRPS	Nonribosomal Peptide Synthetase
OD	Optical Density
OGRI	Overall Genome Relatedness Index
PDA	Potato and Dextrose Agar
PGPB	Plant Growth-Promoting Bacteria
PGPT-Pred	Plant Growth Promotion Trait Prediction
PI	Production Index
PKS	Polyketide Synthase
PLaBAse	Plant-associated Bacteria web resource
PSE	Phosphate Solubilization Efficiency
RAST	Rapid Annotation Subsystem Category
rRNA	Ribosomal RNA
RPB2	second largest RNA polymerase subunits
s.d.H_2_O	Sterile Distilled Water
SD	Standard Deviation
SI	Solubilization Index
SPE	Siderophore Production Efficiency
TEF1	Translation Elongation Factor 1-α
TYGS	Type Strain Genome Server
